# Revolutionising osseous biopsy: the impact of artificial intelligence in the era of personalized medicine

**DOI:** 10.1093/bjr/tqaf018

**Published:** 2025-01-29

**Authors:** Amanda Isaac, Michail E Klontzas, Danoob Dalili, Asli Irmak Akdogan, Mohamed Fawzi, Giuseppe Gugliemi, Dimitrios Filippiadis

**Affiliations:** School of Biomedical Engineering and Imaging Sciences, King’s College London, 100 Lambeth Palace Rd, London SE1 7AR, United Kingdom; Department of Radiology, School of Medicine, University of Crete, Heraklion, Crete, P.C. 71003, Greece; Southwest London Elective Orthopaedic Centre, Epsom and St Helier Hospitals, Surrey, London SM5 1AA, United Kingdom; Ataturk Training and Research Hospital, Izmir Katip Çelebi University, Izmir, Turkey; Department of Radiology, National Hepatology and Tropical Research Institute, Cairo, Egypt; Università Degli Studi di Foggia, Foggia, Italy; 2nd Department of Radiology, University General Hospital “ATTIKON”, Medical School, National and Kapodistrian University of Athens, 12462 Athens, Greece

**Keywords:** artificial intelligence, osseous biopsy, personalized medicine, bone diagnostics, machine learning, precision healthcare

## Abstract

In a rapidly evolving healthcare environment, artificial intelligence (AI) is transforming diagnostic techniques and personalized medicine. This is also seen in osseous biopsies. AI applications in radiomics, histopathology, predictive modelling, biopsy navigation, and interdisciplinary communication are reshaping how bone biopsies are conducted and interpreted. We provide a brief review of AI in image- guided biopsy of bone tumours (primary and secondary) and specimen handling, in the era of personalized medicine. This article explores AI’s role in enhancing diagnostic accuracy, improving safety in biopsies, and enabling more precise targeting in bone lesion biopsies, ultimately contributing to better patient outcomes in personalized medicine. We dive into various AI technologies applied to osseous biopsies, such as traditional machine learning, deep learning, radiomics, simulation, and generative models. We explore their roles in tumour-board meetings, communication between clinicians, radiologists, and pathologists. Additionally, we inspect ethical considerations associated with the integration of AI in bone biopsy procedures, technical limitations, and we delve into health equity, generalizability, deployment issues, and reimbursement challenges in AI-powered healthcare. Finally, we explore potential future developments and offer a list of open-source AI tools and algorithms relevant to bone biopsies, which we include to encourage further discussion and research.

## Introduction

Percutaneous image-guided bone biopsies are invasive but safe and accurate procedures. Biopsy is used to diagnose a range of bone-related conditions, including infection, primary and secondary bone tumours. The biopsy involves extracting a small sample of bone tissue, which is subsequently analysed under a microscope or subjected to a series of testing. Traditionally, biopsy accuracy has depended heavily on the radiologist identifying a lesion as aggressive thereby warranting further characterization on radiological images. This is followed by discussion in a tumour-board meeting, biopsy which is sent for pathology examination to guide further clinical decision making.

Precision medicine customizes treatment to each patient based on individual biology using available information from clinical, morphological, cellular, molecular, and genetic tests. Personalized datasets from multimodal, multiparametric data include molecular omics data (genomics, transcriptomics, proteomics, metabolomics), pathomics, and radiomics. Such large and heterogenous big data allow development of artificial intelligence (AI) algorithms, to enable statistical correlation, predictive modelling, and targeted planning. Screening programs have led to an increased number of patients getting biopsies to investigate incidental lesions, or for cancer detection earlier than previously diagnosed. Advances in antimicrobials and growing concerns of drug-resistance necessitate obtaining samples for culture and sensitivity, microbiome testing, and personalized treatment strategies. The ongoing increase in the extent of biopsy requests worldwide increases the need to optimize biopsy and tests requests, biopsy procedures, and specimen handling.

Advanced research in AI utilities in radiology have explored application related to diagnosis, assessment of the risk of local, regional, and distant metastasis as well as prediction of likely location of regional and distant metastasis (including bone metastasis), prediction of response to treatment and survival. Studies investigating the role of AI in diagnosis have assessed segmentation of tumours on various modalities, role of radiomics in combining genomics and texture analysis of the tumours to differentiate between high grade from intermediate or low grade tumours (eg, chondral lesions and giant cell tumours), differentiating benign form malignant tumours, as well as for planning biopsy trajectories and improving decision making on biopsy locations within the tumours—to avoid necrotic areas and ensure focal cellular representation in heterogenous lesions.

## AI-enabled histopathology

Histopathology, the study of tissue samples under a microscope, has long been the gold standard for diagnosing bone diseases. However, traditional histopathological analysis is time-consuming, labour-intensive, with potential misdiagnosis in complex cases, and time delays in reaching a conclusive diagnosis.^1^ Diagnosis is also subject to variability between different pathologists due to recognized inter-reader variability.^2^^,^^3^AI-enabled assessment of bone marrow cellularity may enable quantitative assessments to be performed more efficiently.^4^ New evidence supports the need to reassess diagnostic pathways for “dedifferentiated tumours”, an entity recognized among a variety of bone and soft tissue neoplasms, including but not limited to chondrosarcoma, parosteal osteosarcoma, and liposarcoma.^1^ AI-assisted histopathology assessment of digitized biopsy slides offers a solution to these challenges by automating the analysis of tissue samples and providing faster, more consistent results.^5^ Digital telepathology paves the way to a more robust pathology service that crosses regional boundaries and allow collaborations to bridge gaps in services and educational needs worldwide.^6^^,^^7^ Additionally, AI systems can process vast amounts of data quickly, making them ideal for high-volume clinical settings where timely diagnosis is critical. A recent study demonstrated large language model (LLM) (ChatGPT-4o) proficiency in analysing pathological images and providing initial diagnoses of bone tumour characteristics is comparable to that of senior pathologists in the Tertiary hospital doctors when compared to junior doctors.^8^^,^^9^ However, there remains inherent limitations to enrolment of ChatGPT and other similar software in clinical practice.^10^ Realizing the potential of generative AI for bone biology will also likely require generating large-scale, high-quality cellular-resolution spatial transcriptomics datasets, improving the sensitivity of current spatial transcriptomics datasets, and thorough experimental validation of model predictions.^11^

Limitations of traditional histopathology pathways:

- Subjectivity: The interpretation of histological slides can vary between pathologists, leading to potential inconsistencies in diagnoses.
- Time-Intensive: Manual analysis of tissue samples is a laborious process, requiring considerable time, especially in complex cases involving multiple biopsies.
- Complexity in rare conditions: Certain bone diseases exhibit subtle or ambiguous histological features, which can lead to misdiagnosis without specialized expertise.
- Shortages in expert bone pathologists: A shortage in expert bone pathologists worldwide may be addressed by creating networks for exchange of information and training as well as exploring alternatives to improve diagnosis of tumours. This would ensure that expert’s input is available to patients in less privileged regions and reduces the risk of burn out to the existing experts who otherwise face excessive increases in workload.^1^^,^^7^^,^^8^

Suggestions for an optimal Pathomics workflow for osseous tumour detection:

1. Image preprocessing: Digitized histological slides are pre-processed to ensure uniformity in image quality. This includes adjusting for colour variations, noise reduction, and normalization.
2. Feature extraction: Image analysis methods, including convolutional neural networks (CNNs), process the images, extracting features such as cell shapes, tissue architecture, and extracellular matrix composition. This enables predicting cell differentiation dynamics, linking molecular and morphological features, and predicting cellular responses to perturbations.^11^
3. Classification/regression: Based on the extracted features, predictive models (eg, CNNs) assign the biopsy sample to diagnostic categories (eg, eosinophilic granuloma, osteomyelitis, benign, osteosarcoma, metastatic) or calculate the risk of the sample to belong to a disease category. This may address growing diagnostic complexity, including ever-expanding cancer protocols and biomarkers.^12^
4. Output interpretation: The AI system provides a diagnostic output along with confidence levels, which could subsequently be reviewed by a pathologist for validation and faster confirmation of the diagnosis.
5. Quality control: Standardized reproducible analysis of large datasets, with appropriate representation of all examined disease conditions, agnostic to other variables allow better quality control of the results. This is particularly relevant in rare disease which are not often encountered in day-to-day practices, in multicentre studies where laboratory settings may introduce bias, as well as in centres where there is paucity of experts to perform regular double reads and calibrations. AI tools can be embedded within a pathology laboratory workflow before or after the diagnosis of the pathologist.
6. Post-market surveillance: AI tools used in bone histopathology should be monitored for performance drifts. Any false negative or false positive results should be reported to the vendor to take appropriate actions and if necessary to re-train the model (based on current regulations this may even affect CE marking of the product).

### AI-based histopathology algorithms

AI algorithms developed for histopathological analysis of bone biopsies were designed to address specific diagnostic challenges. These algorithms utilize a variety of deep learning techniques, including CNNs, recurrent neural networks (RNNs), and generative adversarial networks (GANs). Validated AI-based histopathology tools include:

#### HistoNet: CNN-based bone tumour classification

HistoNet is a deep learning-based algorithm developed for the classification of bone tumours in digitized histopathological slides.^13^ The model is trained on a large dataset of bone biopsy images, with each image labelled by pathologists. HistoNet uses a CNN architecture to extract features such as cell morphology, tissue density, and matrix composition, used to classify the tumour as benign, malignant, or metastatic.

Key features:

- Ability to classify multiple tumour types, including osteosarcoma, chondrosarcoma, and bone metastases.
- Integrates with digital pathology platforms for seamless clinical use.^14^^,^^15^
- Provides confidence scores for each classification, allowing pathologists to review AI-generated diagnoses.
- Enables creation of academic and educational databases.^16^

#### DeepPath: AI-assisted histopathology for bone marrow biopsies and osseous metastasis

DeepPath is an AI platform designed for the analysis of bone marrow biopsies, particularly in diagnosing haematological malignancies and lung metastasis that affect bone health.^17^ The platform utilizes a combination of CNNs and RNNs to analyse the complex histological features of bone marrow, such as cellularity and fibrous matrix patterns.

Key features:

- Capable of detecting haematopoietic disorders such as leukaemia and multiple myeloma as well as lung metastasis.^18^
- Provides visual explanations for AI-driven diagnoses, highlighting regions of interest on biopsy slides.
- Supports integration with electronic medical records (EMR) systems for automated reporting.

#### Inferring super-resolution tissue architecture

Developed by researchers at the Perelman School of Medicine at the University of Pennsylvania, who believe they can help clinicians diagnose and better treat cancers that might otherwise go undetected.^19^

Key features:

- Capable of automatically detecting critical anti-tumour immune formations called “tertiary lymphoid structures”, whose presence correlates with a patient’s likelihood of survival and favourable response to immunotherapy.
- Provides further insight into the novel applications of field of spatial transcriptomics, a relatively new field used to map gene activities within the space of tissues.
- Enables rapid analysis of large cohorts of pathological specimens. When examining a breast cancer dataset the team used, iStar (inferring super-resolution tissue architecture) finished its analysis in just 9 min. By contrast, the best competitor AI tool took more than 32 h to produce similar analysis. That meant that iStar was 213 times faster. iStar can therefore be applied to a large number of samples, which is critical in large-scale biomedical studies and reliably offer objective standardized evaluation in multicentre studies

## AI-enabled radiology

### Accurate tumour segmentation by tumour-normal tissue interface localization

It is important to determine the correct area for the biopsy to be successful. Advanced imaging techniques such as MRI, CT, and PET can reveal good quality details of bone and soft tissues. However, it may not always be easy to determine tumour boundaries, especially in infiltrative lesions. AI can localize the normal and tumour tissue interfaces, allowing for more precise identification of tumour margins and increased biopsy accuracy.^20^ CNNs are excellent at segmenting medical images by learning complex patterns that distinguish tumour tissue from normal bone. A representation of available AI models is summarised in Table 1. CNNs identify nuanced features in imaging data, such as intensity changes that can indicate tumour boundaries even in infiltrative lesions since their architecture is layered. Volumetric assessments may also aid in rapid evaluation of tumour response to therapies, as well as locoregional space-occupying sequelae on neighbouring structures.^21^

The U-Net architecture, which is based on the CNN, has gained popularity for medical image segmentation. U-Net effectively highlights tumour edges by capturing both high- and low-level features. This makes it particularly useful for identifying indefinite boundaries between bone tumours and surrounding tissues. Studies have shown that U-Net’s architecture provides accurate segmentation while reducing manual segmentation time and subjectivity. This helps enhancing the accuracy of diagnosis, treatment planning, and disease monitoring. Also, U-Net has a promising role of supporting precise biopsy and helping avoid normal tissue.

Supervised edge-attention guidance segmentation network (SEAGNET) incorporates spatial attention mechanisms to improve boundary detection, advancing this capability in challenging cases. SEAGNET delineates the scattered edges of tumors by focusing on the most diagnostically relevant areas, achieving high Dice similarity scores and improved segmentation accuracy.^22^ This technology allows radiologists to more confidently define tumor margins and ultimately increasing biopsy sensitivity.

### Radiomics in osseous biopsy

Radiomics is an emerging field where quantitative features are extracted from medical imaging, transforming images into datasets for high-fidelity region-of-interest analysis.^23^ Quantitative radiomics data include first order, texture and shape features as well as high-order features produced by image filtering (including wavelet, Laplacian of Gaussian, Gabor filters) to provide more detailed perspective of the internal structures of bones and adjacent tissues.^24^ Radiomics data can be extracted either with standardized algorithms and image filters (handcrafted radiomics) or with the use of CNNs (deep radiomics). Handcrafted radiomics methods are applied in the vast majority of publications due to the explainable nature of the features, whereas the black box nature of deep radiomics does not favour their wide utilization.^25^ Radiomics supersedes the human’s eye ability to discern the entire grey scale enabling the detection of tissue-specific patterns and providing high-dimensional data that can be used for the construction of predictive models.

**Figure 1.**
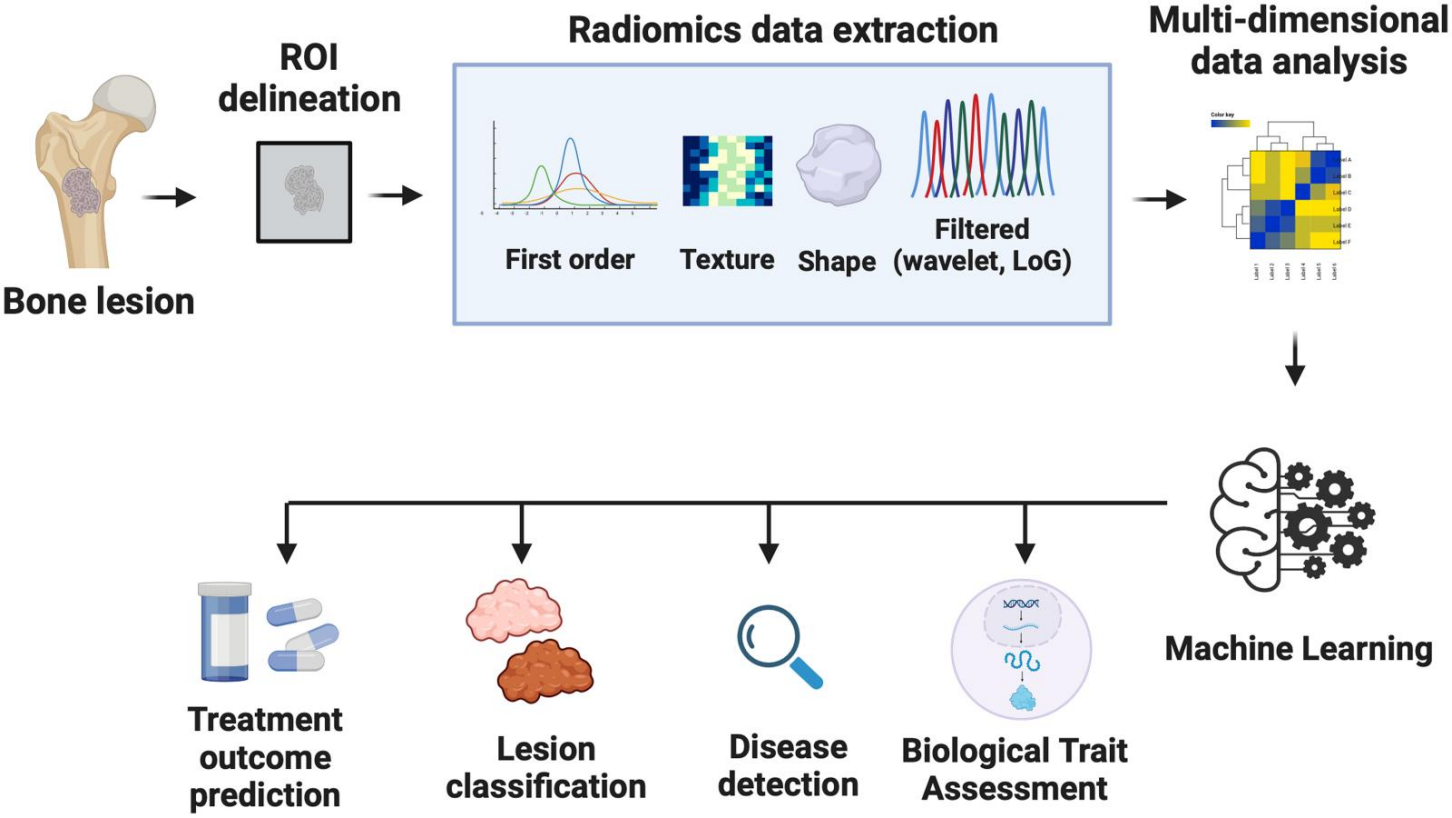
Diagram illustrating the application of radiomics on bone biopsy. Lesions are identified on imaging, regions of interest (ROI) are delineated and radiomics features are extracted (either handcrafted or deep radiomics) and the dataset is analysed to extract useful features for machine learning model development. These models can perform a series of tasks ranging from disease detection to treatment outcome prediction (created with biorender.com).

In the context of bone analysis, radiomics have found uses in the analysis of osteoblastic or osteolytic metastases, the evaluation of the bone marrow in the context of multiple myeloma, the differentiation between benign and malignant chondrogenic bone tumours and the prediction of response to chemotherapy (Figure 1). Radiomics analysis has been found capable to distinguish between bone islands and osteoblastic metastases, performing better than inexperienced human readers.^26^ It has been also used for the differentiation between osteolytic metastases and multiple myeloma lesions achieving similar performance to musculoskeletal radiologists.^27–29^

The capacity of radiomics to identify texture patterns allows the differentiation between tissue types. Nonetheless, classification tasks require coupling of radiomics with machine learning algorithms suitable for tabular data. Such machine learning-enabled radiomics analysis is commonly used when radiomics are tasked to provide solutions to binary classification problems such as distinguishing metastases from primary bone lesions or differentiating between benign and malignant lesions such as the differentiation between atypical cartilaginous tumours and chondrosarcomas. The segmentation part of radiomics analysis can be augmented with the use of deep learning-based segmentation for the step that always precedes feature extraction. Such combined approaches have allowed automatic scanning of the skeleton for metastases for subsequent feature extraction and downstream analysis.

Radiomics analysis has been employed for the evaluation of multiple myeloma lesions, either by probing single lytic myeloma foci or by performing whole-marrow segmentation and analysis of disease load. Wennmann et al have used deep learning for the automated segmentation of the bone marrow in whole body MRI subsequently extracting whole-marrow radiomics data.^30^ This dataset successfully predicted biopsy results including high-risk cytogenetic status, and chromosomal aberrations.^30–32^ This approach is particularly useful for the comprehensive assessment of smouldering myeloma, enabling non-invasive re-staging, biopsy result, and therapy response prediction. Being able to predict genetic/biological traits of the disease such as the presence of chromosomal aberrations is extremely important. This has been also demonstrated in cases of primary bone tumours, where the expression of p53 and VEGF can be predicted solely by means of radiomics analysis.^33^ Similarly, the expression of EGFR in osseous metastases can be predicted by means of radiomics analysis.^34^ Probing bone lesions with radiomics has been also shown to successfully predict response to certain treatments such as the response of osteosarcoma and multiple myeloma to chemotherapy.^19^^,^^35^^,^^36^

### Radiomics-based multimodal data integration

The high-dimensional nature of radiomics datasets renders them ideal candidates for the integration with other types of high-dimensional data, to extract information about the multi-level function of human tissues. It has been postulated that the combination of information obtained from multiple layers of tissue function (eg, genes, transcripts, proteins, metabolites) can allow disease subtyping, biomarker identification, and the phenotyping of complex disease states.^37^ Since radiomics represent the combination of all layers of tissue function that lead to a final imaging phenotype, the integration with other types of omics data has led to the construction of complex multivariate signatures that enable disease phenotyping by taking into account both the imaging appearance and the underlying biology of the disease. In the context of bone disease, radiogenomics signatures have enabled the prediction of the response to chemotherapy and the development of metastatic disease in cases of osteosarcoma.^38^ As shown in other types of malignancies, radiomics can be also integrated with pathomics (multivariate data derived from traditional histology) to predict surgical resection outcomes, offering superior performance compared to pathomics-only or radiomics-only methods. Future integration of radiomics with other types of biological data could increase the predictive capacity of radiomics models while indicating links between biology and imaging traits (see Table 1).

**Table 1.** AI applications in bone tumours: an overview.

Title	DOI	Application	AI techniques	Number of patients and nature of tumour (primary or secondary, and origin if metastatic)	Imaging modality evaluated	Dataset type
International validation of the Skeletal Ontology Research Group (SORG) machine-learning algorithm for predicting the survival of patients with extremity metastases undergoing surgical treatment	10.1097/corr.0000000000001969	Predicting the 90-day and 1-year mortality or survival of patients with extremity metastases undergoing surgical treatment in Taiwan	Multiple traditional machine-learning algorithms, assessed by the skeletal oncology research group machine-learning algorithms (sorg-mlas)	356 Taiwanese patients, 349/356 of Han Chinese descent. The most common primary tumours were lung cancer (33% [116 of 356]) and breast cancer (16% [58 of 356])	Multimodal	Histology from surgical specimens
Establishment and validation of a machine learning prediction model based on big data for predicting the risk of bone metastasis in renal cell carcinoma patients	10.1155/2022/5676570	Predicting the risk of bone metastasis in renal cell carcinoma patients	Traditional machine learning	40 355	Multimodal	Histopathology, clinicopathological features, serological markers demographic data, and follow-up data
Diagnostic performance of deep learning models for detecting bone metastasis on whole-body bone scan in prostate cancer	10.1007/s00259-021-05481-2	Detecting bone metastasis on whole-body bone scan in prostate cancer	Deep learning	9113 consecutive bone scans (5342 prostate cancer patients)	Whole-body bone scans Whole-body bone scans were performed 3-4 h after intravenous injection of 99mtc-diphosphono-1,2-propanodicarboxylic acid (99mtc-dpd; 3976 scans) or 99mtc-hydroxymethylene diphosphonate (99mtc-hpd; 5157 scans)	Serum PSA at scan time (ng/mL) Serum testosterone at scan time Time since diagnosis Local therapy before scan • prostatectomy, radiation treatment or systemic therapy before scan, androgen deprivation therapy or chemotherapy
Multitask deep learning for segmentation and classification of primary bone tumours on radiographs	10.1148/radiol.2021204531	Segmentation and classification of primary bone tumours	Deep learning	934 patients, which included 667 benign bone tumours and 267 malignant bone tumours	Plain radiographs	Benign or malignant bone tumours were diagnosed in all patients by using the histopathologic findings as the reference standard
Deep learning on MR images for diagnosis of lung cancer spinal bone metastasis	10.1155/2021/5294379	Diagnosis of lung cancer spinal bone metastasis	Deep learning	87	MRI	Pathology specimens of bone biopsies and lung cancer specimens
Using deep learning for quantification of cellularity and cell lineages in bone marrow biopsies and comparison to normal age-related variation	10.1016/j.pathol.2021.07.011	Quantification of cellularity and cell lineages in bone marrow biopsies and comparison to normal age-related variation. Assessment of cellularity, myelopoiesis and megakaryocytes by age	Deep learning	130	Multimodality	Digital image analysis of bone marrow biopsies of patients and controls
Machine learning of genomic features in organotrophic metastases stratifies progression risk of primary tumours	10.1038/s41467-021-27017-w	To assess metastatic risks of primary tumours. Identify metastasis-featuring primary (mfp) tumours, a subset of primary tumours with genomic features enriched in metastasis and demonstrate their higher metastatic risk and shorter disease-free survival. In addition, we identify genomic alterations associated with organ-specific metastases and employ them to stratify patients into various risk groups with propensities toward different metastatic organs	Traditional Machine learning—METAnet	32 176 primary and metastatic cancer patients including bone primary and metastasis	–	Clinical and genetic sequencing against histological grading
MRI radiomics-based machine learning classification of atypical cartilaginous tumour and grade II chondrosarcoma of long bones	10.1016/j.ebiom.2021.103757	Classification of atypical cartilaginous tumour and grade II chondrosarcoma of long bones	Radiomics based machine learning	158	MRI	Surgically treated and histology-proven cartilaginous bone tumours
Diffusion-weighted radiomics of spine bone tumours: feature stability and machine learning-based classification performance	10.1007/s11547-022-01468-7		Diffusion-weighted and T2WMRIT2Wi MRI radiomics machine learning	101 patients with histology-proven spine bone tumour (22 benign; 38 primary malignant; 41 metastatic)	MRI	Histological specimens and clinical diagnosis
Automated prediction of the neoadjuvant chemotherapy response in osteosarcoma with deep learning and an iMRIMRI-based radiomics nomogram	10.1007/s00330-022-08735-1	Prediction of the neoadjuvant chemotherapy response in osteosarcoma	Radiomics based machine learning	144 osteosarcoma patients	MRI	Histological specimens and clinical diagnosis
Development and evaluation of machine learning models based on X-ray radiomics for the classification and differentiation of malignant and benign bone tumours	10.1007/s00330-022-08764-w	X-ray based radiomics for the classification and differentiation of malignant and benign bone tumours	Radiomics based machine learning	880 patients (age 33.1 ± 19.4 years, 395 women) diagnosed with malignant (*n* = 213, 24.2%) or benign (*n* = 667, 75.8%) primary bone tumours	Plain radiographs	Histological specimens and clinical diagnosis including demographics
Combining deep learning and radiomics for automated, objective, comprehensive bone marrow characterization from whole-body MRI: a multicentric feasibility study	10.1097/rli.0000000000000891	Automated, objective, comprehensive bone marrow characterization from whole-body MRIiMRI using deep learning and radiomics	Combined deep learning and radiomics	106 WB-MRI	MRI—whole body mMRI	Histological specimens and clinical diagnosis including demographics
Machine learning models for the diagnosis and prognosis prediction of high-grade B-cell lymphoma	10.3389/fimmu.2022.919012	Diagnosis and prognosis prediction of high-grade B-Cell lymphoma	Traditional machine learning	187 patients with aggressive mature B-cell lymphomas	PET-CT	Histological specimens and clinical diagnosis including survival, chemotherapeutic regimens and serological tests including Epstein-Barr virus-encoded small nuclear RNA (EBER) and Ann Arbor staging
A scoring system for predicting neoadjuvant chemotherapy response in primary high-grade bone sarcomas: a multicentre study	10.1111/os.13469	Predicting neoadjuvant chemotherapy response in primary high-grade bone sarcomas including regional and distant bone metastasis	Several machine learning models, including logistic regression, decision trees, support vector machines, and neural networks, were used to classify the chemotherapy responses	322 patients	X-ray, CT, contrast-enhanced magnetic resonance (MR), and positron emission tomography-CT (PET-CT)	Histological specimens and clinical diagnosis including serological tests
Detection of bone metastases on bone scans through image classification with contrastive learning	10.3390/jpm11121248	Detection of bone metastases on bone scans	Traditional machine learning	37 427 sets of images of 19 041 patients	Routine whole-body scans were performed 2-4 h after intravenous administration of 20 mCi of 99mTc-labeled MDP w	Histopathology
Deep learning based automated diagnosis of bone metastases with SPECT thoracic bone images	10.1038/s41598-021-83083-6	Automated diagnosis of bone metastases with SPECT thoracic bone images	Deep learning	251 patients, 346 datasets, diagnosed with normal (*n* = 166, ≈ 66%) and metastasis (*n* = 85, ≈ 34%)	Whole-body SPECT bone scan	Histopathology and clinical
Bone tumour necrosis rate detection in few-shot X-rays based on deep learning	10.1016/j.compmedimag.2022.102141	Bone tumour necrosis rate detection on X-rays	Traditional machine learning	119	Plain radiographs	Histopathology and clinical
Automated bone tumour segmentation and classification as benign or malignant using computed tomographic imaging	10.1007/s10278-022-00771-z	Classification as benign or malignant bone tumours in using computed tomographic imaging of femora		84	CT	Histopathology and clinical
An accurate prediction of the origin for bone metastatic cancer using deep learning on digital pathological images	10.1016/j.ebiom.2023.104449	Predicting origin for bone metastatic cancer using deep learning-based pathology	Deep learning	1041	–	Pathology slides and retrospective datasets
MarrowQuant 2.0: a digital pathology workflow assisting bone marrow evaluation in experimental and clinical haematology	10.1016/j.modpat.2022.100088	Bone marrow quantitative assessment of cellularity and integrated workflow pathway	MarrowQuan and QuPath software	250 BM specimens from iliac crest trephines	–	Pathology slides and retrospective datasets
An automated pipeline for differential cell counts on whole-slide bone marrow aspirate smears	10.1016/j.modpat.2022.100003	Cellular count on pathology aspirates of bone marrow	Traditional machine learning	396 048	–	Pathology slides and retrospective datasets
Prognosis of thyroid carcinoma patients with osseous metastases: a SEER-based study with machine learning	10.1007/s12149-023-01826-z	Assess the prognosis of thyroid carcinoma patients with osseous metastases	Traditional machine learning	579	–	Demographics, histopathology, TNM staging, surgical, and radiotherapy notes
Differentiation of bone metastasis in elderly patients with lung adenocarcinoma using multiple machine learning algorithms	10.1177/10732748231167958	Differentiation of bone metastasis in elderly patients with lung adenocarcinoma	Multiple machine learning algorithms	27 627	–	Demographics, histopathology, TNM staging, surgical and radiotherapy notes
Diagnostic value of machine learning-based computed tomography texture analysis for differentiating multiple myeloma from osteolytic metastatic bone lesions in the peripheral skeleton	10.1007/s00256-023-04333-4	Computed tomography texture analysis for differentiating multiple myeloma from osteolytic metastatic bone lesions in the appendicular skeleton	Traditional machine learning	172 patients with multiple myeloma (*n* = 70) and osteolytic metastatic bone lesions (*n* = 102) in the peripheral skeleton	CT	Histopathology and clinical
Radiomics analysis of bone marrow biopsy locations in [18F]FDG PET/CT images for measurable residual disease assessment in multiple myeloma	10.1007/s13246-023-01265-0	Bone marrow biopsy locations in [18F]FDG PET/CT images for measurable residual disease assessment in multiple myeloma	Traditional machine learning	39	Whole body [^18^F]FDG PET images	Histopathology and clinical
Predicting patterns of distant metastasis in breast cancer patients following local regional therapy using machine learning	10.3390/genes14091768	Predicting patterns of distant metastasis in breast cancer patients following local regional therapy	Traditional machine learning	175	Standard multimodal imaging	Histopathology and clinical
A machine learning-based model for clinical prediction of distal metastasis in chondrosarcoma: a multicentre, retrospective study	10.7717/peerj.16485	Clinical prediction of distal metastasis in chondrosarcoma: a multicentre, retrospective study	Traditional machine learning	1385	Standard multimodal imaging	Demographics, histopathology, TNM staging, surgical, and radiotherapy notes
Deep learning-based detection and classification of bone lesions on staging CT in prostate cancer: a development study	10.1016/j.acra.2024.01.009	Detection and classification of bone lesions on staging CT in prostate cancer	Deep learning	297 staging CT scans (81 metastatic) with 4601 benign and 1911 metastatic lesions in PCa patients.	CT	Histopathology and clinical
X-rays radiomics-based machine learning classification of atypical cartilaginous tumour and high-grade chondrosarcoma of long bones	10.1016/j.ebiom.2024.105018	X-rays radiomics-based machine learning classification of atypical cartilaginous tumour and high-grade chondrosarcoma of long bones	Traditional machine learning	150	Plain radiographs	Histopathology and clinical
A radiograph-based deep learning model improves radiologists' performance for classification of histological types of primary bone tumours: a multicentre study	10.1016/j.ejrad.2024.111496	Improving radiologists' performance for classification of histological types of primary bone tumours on plain radiographs	Deep learning	878	Plain radiographs	Histopathology and clinical
Development and validation of a machine learning model for bone metastasis in prostate cancer: based on inflammatory and nutritional indicators	10.1016/j.urology.2024.05.027	Bone metastasis in prostate cancer, based on inflammatory and nutritional indicators	Traditional machine learning	627	Histopathology and laboratory data	Gleason scores from histopathology and clinical data
Sarcoma classification by DNA methylation profiling	10.1038/s41467-020-20603-4	Sarcoma classification by DNA methylation profiling	A classification tool, sarcoma classifier, using a random forest machine learning classification algorithm	1077	Histopathology: DNA methylation-based categorization	Methylomics from traditional histopathology and clinical

### AI-enabled bone biopsy navigation and guidance

Accurate targeting of a lesion is crucial to ensure a representative sample is obtained. Heterogenous lesion should be biopsies from as many regions as feasible to gain a more comprehensive understanding of the extent of disease. Biopsy trajectories are planned to avoid neurovascular bundles and adjacent vital structures, to minimize morbidity. A sampling error of the biopsy material that contains reactively altered tissue must always be considered.^39^ If in doubt, repeat sampling must be considered. Irrespective of biopsy method, it is essential to avoid haematomas, foci of adjacent collections, and contamination of neurovascular structures or joints. All adjacent tissue considered to be contaminated must be resected afterwards if malignancy is confirmed.

For suspected primary sarcoma, biopsy procedures should be performed at a specialist sarcoma referral centre following multidisciplinary bone tumour-board discussions with the surgeons who will carry out definitive tumour resection. Larger resections and amputations due to inappropriate needle biopsy technique, where limb salvage could have been possible, have been described. The biopsy site should be marked with a skin tattoo allow its identification at time of surgery following neoadjuvant chemotherapy.^40^

**Figure 2.**
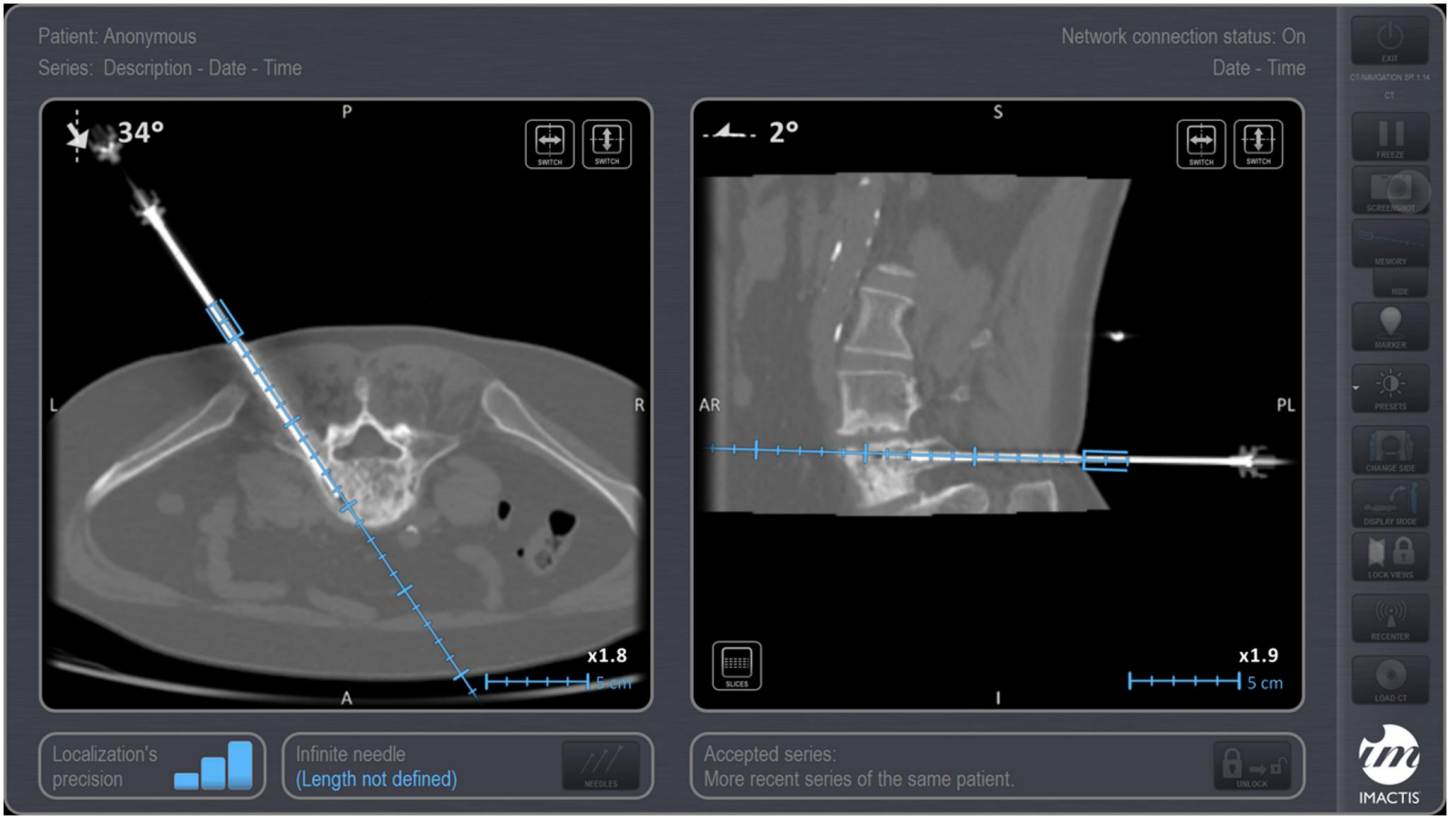
A 51 y-o female breast cancer patient with a mixed L5 lesion underwent percutaneous biopsy under CT guidance using an electromagnetic navigation system. Notice the accuracy and lack of Euclidian error between the planned (blue line) approach as selected in the planning screenshot and the biopsy needle as placed in the final control screenshot.

**Figure 3.**
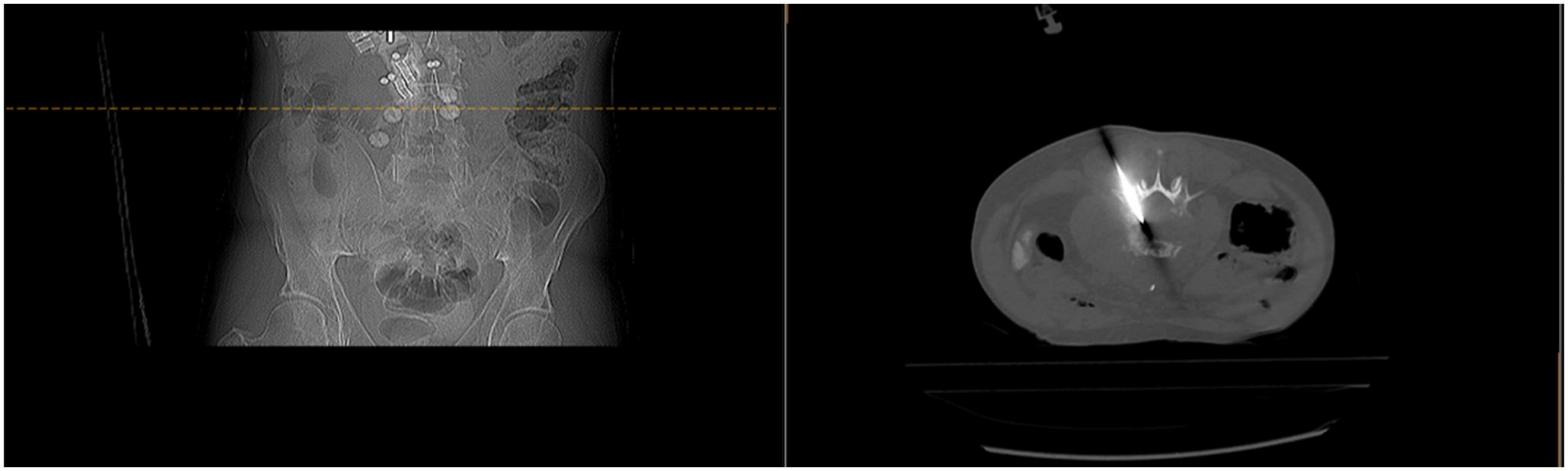
Robotic assisted biopsy of an L4 lesion under CT guidance using a table-mounted robotic system (micromate/interventional systems).

With regards to navigation, current guidance systems for bone biopsies include fusion imaging and needle tracking, electromagnetic navigation (Figure 2) as well as robotics (Figure 3); numerous studies have shown efficacy and safety of percutaneous CT-guided bone biopsies in patients with cancer using such systems.^41^ Garnon et al utilized combined fusion imaging and needle tracking under ultrasound guidance to target bone lesions without cortical disruption in seven patients reporting that the approach seems technically feasible, provided the patient and lesion selection is appropriate.^42^ Groetz et al evaluated a table-mounted robotic device in an animal study as well as in three patients reporting accurate and simple stereotactic bone biopsies, avoiding the need for needle readjustment.^43^ In their study, Witkowska et al retrospectively evaluated 39 patients who underwent 40 bone biopsies in various skeletal locations using a patient-mounted robotic system with steering capabilities reporting high technical success, adequate diagnostic yield, and favourable safety profile.^44^

These systems focus more on navigation itself offering reduced intervention time and radiation exposure along with accurate alignment to trajectory and needle angulation but with limited use of AI upon the procedural steps (figure—figure). On the other hand, available mixed or augmented reality devices and software have been tested to visualize ultrasound data and spatial navigation information facilitating thus faster and precise biopsy sessions.^45^^,^^46^ These experimental studies report that such approaches could be beneficial for physicians at any level of expertise.

### AI-enabled reporting and workflow planning

To our knowledge, AI-enhanced workflows have not yet been integrated formally into clinical bone tumour pathways—including those for metastasis. Several studies have explored its potential in simulated, retrospective settings, demonstrating significant cost and time savings, and increased accuracy.^47^ However, there remains logistical challenges that must be addressed before robust adoption of AI into clinical practice is deemed achievable. Limitations to engagement of AI systems in practice, such as willingness to use, effective integration, and AI impact on training and supervision.^48^ When approached, patients favoured clinicians over AI in most clinical tasks and strongly preferred an application of AI if with physician supervision.^49^ However, patients acknowledged that AI could help physicians integrate the most recent scientific evidence into medical care. Patients have also requested that application of AI in medicine should be disclosed and controlled to ensure clinical experts are guiding the workflows to protect patient interests and meet ethical standards.^49^ Several groups published recommendations for best practice integration of AI into clinical worksflows (not exclusive to bone tumours). Those include: (1) building safe and trustworthy systems; (2) developing validation, verification, and certification processes for AI-CDS systems; (3) providing a means of safety monitoring and reporting at the national level; and (4) ensuring that appropriate documentation and end-user training are provided.^50^

### Potential benefits of integrating AI into osseous biopsies

AI can significantly augment traditional diagnostic techniques by providing faster, more reliable results by integrating multiparametric, multivariate parameters specific to each patient, as well as providing additional information with radiomics texture analysis that are otherwise not feasible to the human eye. Potential benefits include helping radiologists to improve their accuracy and confidence in their clinical interpretation of radiological studies, improve targeting of tumours to achieve higher yield of biopsies, reducing the need to biopsy benign lesions if accurately diagnosed as such, improve timing of re-biopsies in tumours where there is a concern for de-differentiation following radiotherapy or resistance to treatments. Stratification algorithms demonstrate superior accuracy compared to standard histological grading with better prognostic value for survival longevity and metastatic risk.^51^ Predictions can be used to formulate scoring systems that allow standardized communication between clinicians and comparison of various therapeutic options to assess efficacy.^52^ Imaging of bone metastasis relies on advanced complex 3D imaging modalities, including CT, MRI, SPECT-CT, and PET. These modalities are not always feasible because of the high cost and long acquisition time and the complex logistics of acquiring positron-emitting agents, particularly in less developed countries or at time of shortages. Segmentation and accurate characterization of tumours can potentially reduce diagnostic errors and improve the speed of diagnosis in busy clinical settings. Recent research studies have assessed the capacity of AI to improve diagnosis of bone metastasis on planar bone scans, which are relatively cheaper and in some settings more widely available.^53^ Several studies assessing performance of the AI algorithms against radiologists have demonstrated performance that surpasses that of radiologic residents or general radiologists and are comparable with that of fellowship-trained radiologists.^54^ This may offer additional support in remote regions where availability of expert oncology radiologists are scarce and may alleviate pressure on busy tumour units as a double read, reducing the risk of burn out to expert radiologists. Xu et al demonstrated AI tools to stratify tumour necrosis rates as an indicator of the effect of chemotherapy, applied on X-ray images at different chemotherapy stages.^55^ Necrosis rate ranges from 0% to 100% and in the medical field 90% is usually considered as a threshold point that is especially useful for the follow-up treatment of bone tumour.^56^ The classification model correlates between the chemotherapy effect over time and its characteristic changes on X-ray images. This has the potential to reduce the need for interval bone biopsies to assess chemotherapy response.

### Limitations of AI in osseous biopsies

Limitations include tardiness in initial adoption due to shortcomings in the existing tools, which hinder their broad integration in the decision-making processes of pathologists. Limitations of evaluating prognostic research related to AI mirror other studies in this patient cohort. There are limitations in the quality and comprehensiveness of large datasets, particularly in rare disease.^57^ In patients presenting with metastatic bone disease (Stage 4 on the TNM score), the overall survival is poor, and many patients are lost to follow up thereby limiting the window for validating predictive models for survival. There remain concerns regarding the long-term patients benefits of integrating AI into day-to-day workflows, generalizability in real-world populations and across all ethnic groups, the robustness of the algorithms in intermediate-grade and undifferentiated or heterogenous tumours.^58^ The use of radiomics is still limited by the small datasets, the manual nature of radiomics analysis that requires relevant expertise, the lack of integration into PACS systems, and the hesitance of radiologists to use methods that they are not entirely familiar with.

To date, only a handful of applications have formal FDA approvals and CE or UK marking and are commercially available. Even the available CE marked products are not backed up by appropriate scientific evidence, reducing the trust of the end-users (pathologists & radiologists) and subsequently the wider use by the MSK tumour MDTs.^59^ Limitations include tardiness in initial adoption due to shortcomings in the existing tools, which hinder their broad integration in the decision-making processes of pathologists. Limitations of evaluating prognostic research related to AI mirror other studies in this patient cohort. In patients presenting with metastatic bone disease (Stage 4 on the TNM score), the overall survival is poor, and many patients are lost to follow up thereby limiting the window for validating predictive models for survival. There remain concerns regarding the long-term patients benefits of integrating AI into day-to-day workflows, generalizability in real-world populations and across all ethnic groups, the robustness of the algorithms in intermediate-grade and undifferentiated or heterogenous tumours.

Limitations also include cost implication associated with the use of novel technology, challenges of survival of most small to intermediate size companies which have not yet been able to secure financial sustainability—despite seemingly promising quality of their software amidst the current financial challenges facing most healthcare models, and last but not least bias against patients in underprivileged parts of the world, where such technology may not be accessible. Last but not least, liability of the users in cases of false negative or false negative diagnoses still remains an important hurdle in the widespread implementation of AI in musculoskeletal oncology and radiology in general. The newly released AI act of the European Union does not cover liability issues and does not yet consider medical imaging as a specific case of medical AI.^60^

### Future directions

Creating a positive culture that enables robust research for the assessment of novel technologies including AI alongside day-to-day practices is required to evaluate accuracy, robustness and reduces the limitations of the algorithms safely and objectively. This enables improvement of AI models on real-world data.

Multicentre trials using federated learning on large datasets may facilitate roll out of AI in patient care. Integrating imaging based radiomics with biological omics (genomics, transcriptomics, proteomics, metabolomics), clinical biomarkers, and demographics significantly increases the performance of radiomics models, allowing extraction of several phenotypes, thereby improving the applicability of the software in clinical practice.^61^

## Conclusion

AI-enabled techniques for bone biopsy have enormous potential to improve practice by reducing errors, enhancing safety, improving reproducibility, and facilitating expert communication, all of which were previously difficult with traditional techniques, and limited to expert specialized units. Innovations of AI applications should be affordable, practical, interoperable, explainable, ethical, generalizable, manageable, auditable, and reimbursable. In the era of personalized medicine good quality specimens from the metastases are crucial both at the time of the primary bone metastatic event and also later in the disease process. In the near future, research might lead to further innovative targeted treatments. The increasing demand of multiple tests of a specimen highlights the need of optimal biopsy target selection, biopsy techniques, and specimen handling. Furthermore, AI will help to categorize and analyse incoming data from clinical files, radiology, biopsy procedures, pathology, and new generation tests for improved outcome in personalized medicine and as a benefit also in individualized medicine.

## References

[1] Kilpatrick SE. Keeping it real: merging traditional and contemporary practices in musculoskeletal pathology: a special issue of neoplastic and non-neoplastic bone and soft tissue pathology. Hum Pathol. 2024;147:1-4. 38556003 10.1016/j.humpath.2024.03.007

[2] Burns J , Wilding CP, R LJ, P HH. Proteomic research in sarcomas—current status and future opportunities. Semin Cancer Biol. 2020;61:56-70. 31722230 10.1016/j.semcancer.2019.11.003 PMC7083238

[3] Ray-Coquard I , Montesco MC, Coindre JM, et al Sarcoma: concordance between initial diagnosis and centralized expert review in a population-based study within three European regions. Ann Oncol. 2012;23:2442-2449. 22331640 10.1093/annonc/mdr610 PMC3425368

[4] Sarkis R , Burri O, Royer-Chardon C, et al MarrowQuant 2.0: a digital pathology workflow assisting bone marrow evaluation in experimental and clinical hematology. Mod Pathol. 2023;36:100088. 36788087 10.1016/j.modpat.2022.100088

[5] Romero D. AI to assess images. Nat Rev Clin Oncol. 2018;15:724. 10.1038/s41571-018-0107-y 30266916

[6] Sohani AR , Sohani MA. Static digital telepathology: a model for diagnostic and educational support to pathologists in the developing world. Anal Cell Pathol (Amst). 2012;35:25-30. 22233701 10.3233/ACP-2011-0032 PMC4605715

[7] Hitchcock CL. The future of telepathology for the developing world. Arch Pathol Lab Med. 2011;135:211-214. 21284440 10.5858/135.2.211

[8] Huang L , Hu J, Cai Q, et al Preliminary discrimination and evaluation of clinical application value of ChatGPT4o in bone tumors. J Bone Oncol. 2024;48:100632. 39310381 10.1016/j.jbo.2024.100632 PMC11414679

[9] Aden D , Zaheer S, Khan S. Possible benefits, challenges, pitfalls, and future perspective of using ChatGPT in pathology. Rev Esp Patol. 2024;57:198-210. 38971620 10.1016/j.patol.2024.04.003

[10] Oon ML , Syn NL, Tan CL, Tan KB, Ng SB. Bridging bytes and biopsies: a comparative analysis of ChatGPT and histopathologists in pathology diagnosis and collaborative potential. Histopathology. 2024;84:601-613. 38032062 10.1111/his.15100

[11] Lu L , Ono N, Welch JD. Linking transcriptome and morphology in bone cells at cellular resolution with generative AI. J Bone Miner Res. 2024;40:20-26. 39303095 10.1093/jbmr/zjae151 PMC11700600

[12] Waqas A , Bui MM, Glassy EF, et al Revolutionizing digital pathology with the power of generative artificial intelligence and foundation models. Lab Invest. 2023;103:100255. 37757969 10.1016/j.labinv.2023.100255

[13] Hoefling H , Sing T, Hossain I, et al HistoNet: a deep learning-based model of normal histology. Toxicol Pathol. 2021;49:784-797. 33653171 10.1177/0192623321993425

[14] Filler TJ , Abele H, Vollmar-Hesse I, Peuker ET. [New paths for Internet generated learning in anatomy]. Ann Anat. 1999;181:499-508. 10560017 10.1016/s0940-9602(99)80034-x

[15] Lun KC , Tan TW, Gopalakrishnakone P, Loh S. Delivering health information databases on World Wide Web at the National University of Singapore. Medinfo. 1995;8 Pt 2:1528. 8591490

[16] Brochhausen C , Winther HB, Hundt C, Schmitt VH, Schömer E, Kirkpatrick CJ. A virtual microscope for academic medical education: the pate project. Interact J Med Res. 2015;4:e11. 25963527 10.2196/ijmr.3495 PMC4443020

[17] Patil PD , Hobbs B, Pennell NA. The promise and challenges of deep learning models for automated histopathologic classification and mutation prediction in lung cancer. J Thorac Dis. 2019;11:369-372. 30962976 10.21037/jtd.2018.12.55 PMC6409244

[18] Coudray N , Ocampo PS, Sakellaropoulos T, et al Classification and mutation prediction from non-small cell lung cancer histopathology images using deep learning. Nat Med. 2018;24:1559-1567. 30224757 10.1038/s41591-018-0177-5 PMC9847512

[19] Zhang D , Schroeder A, Yan H, et al Inferring super-resolution tissue architecture by integrating spatial transcriptomics with histology. Nat Biotechnol. 2024;42:1372-1377. 38168986 10.1038/s41587-023-02019-9 PMC11260191

[20] Sabeghi P , Kinkar KK, Castaneda GDR, et al Artificial intelligence and machine learning applications for the imaging of bone and soft tissue tumors. Front Radiol. 2024;4:1332535. 39301168 10.3389/fradi.2024.1332535 PMC11410694

[21] Sachpekidis C , Enqvist O, Ulén J, et al Artificial intelligence-based, volumetric assessment of the bone marrow metabolic activity in [(18)F]FDG PET/CT predicts survival in multiple myeloma. Eur J Nucl Med Mol Imaging. 2024;51:2293-2307. 38456971 10.1007/s00259-024-06668-z PMC11178614

[22] Zhan X , Liu J, Long H, et al An intelligent auxiliary framework for bone malignant tumor lesion segmentation in medical image analysis. Diagnostics (Basel). 2023;13. 10.3390/diagnostics13020223 PMC9858155 36673032

[23] Papanikolaou N , Matos C, Koh DM. How to develop a meaningful radiomic signature for clinical use in oncologic patients. Cancer Imaging. 2020;20:33. 32357923 10.1186/s40644-020-00311-4 PMC7195800

[24] van Timmeren JE , Cester D, Tanadini-Lang S, Alkadhi H, Baessler B. Radiomics in medical imaging-"how-to" guide and critical reflection. Insights Imaging. 2020;11:91. 32785796 10.1186/s13244-020-00887-2 PMC7423816

[25] Scapicchio C , Gabelloni M, Barucci A, Cioni D, Saba L, Neri E. A deep look into radiomics. Radiol Med. 2021;126:1296-1311. 34213702 10.1007/s11547-021-01389-x PMC8520512

[26] Hong JH , Jung J-Y, Jo A, et al Development and validation of a radiomics model for differentiating bone islands and osteoblastic bone metastases at abdominal CT. Radiology. 2021;299:626-632. 33787335 10.1148/radiol.2021203783

[27] Lee S , Lee S-Y, Kim S, et al Differentiating multiple myeloma and osteolytic bone metastases on contrast-enhanced computed tomography scans: the feasibility of radiomics analysis. Diagnostics (Basel). 2023;13:755. doi: 10.3390/diagnostics13040755. 36832243 PMC9955828

[28] Gitto S , Cuocolo R, Annovazzi A, et al CT radiomics-based machine learning classification of atypical cartilaginous tumours and appendicular chondrosarcomas. EBioMedicine. 2021;68:103407. 34051442 10.1016/j.ebiom.2021.103407 PMC8170113

[29] Xu Y , Meng C, Chen D, Cao Y, Wang X, Ji P. Improved localization and segmentation of spinal bone metastases in MRI with nnUNet radiomics. J Bone Oncol. 2024;48:100630. 39281712 10.1016/j.jbo.2024.100630 PMC11399709

[30] Wennmann M , Ming W, Bauer F, et al Prediction of bone marrow biopsy results from MRI in multiple myeloma patients using deep learning and radiomics. Invest Radiol. 2023;58:754-765. 37222527 10.1097/RLI.0000000000000986

[31] Wennmann M , Klein A, Bauer F, et al Combining deep learning and radiomics for automated, objective, comprehensive bone marrow characterization from whole-body MRI: a multicentric feasibility study. Invest Radiol. 2022;57:752-763. 35640004 10.1097/RLI.0000000000000891

[32] Klontzas ME , Triantafyllou M, Leventis D, et al Radiomics analysis for multiple myeloma: a systematic review with radiomics quality scoring. Diagnostics (Basel). 2023;13:2021. doi: 10.3390/diagnostics13122021. 37370916 PMC10296889

[33] Wang Q , Zhang Y, Zhang E, et al A multiparametric method based on clinical and CT-based radiomics to predict the expression of p53 and VEGF in patients with spinal giant cell tumor of bone. Front Oncol. 2022;12:894696. 35800059 10.3389/fonc.2022.894696 PMC9253421

[34] Fan Y , Dong Y, Sun X, et al Development and validation of MRI-based radiomics signatures as new markers for preoperative assessment of EGFR mutation and subtypes from bone metastases. BMC Cancer. 2022;22:889. 35964032 10.1186/s12885-022-09985-4 PMC9375915

[35] Wu Z , Bian T, Dong C, et al Spinal MRI-based radiomics analysis to predict treatment response in multiple myeloma. J Comput Assist Tomogr. 2022;46:447-454. 35405690 10.1097/RCT.0000000000001298

[36] Chen H , Zhang X, Wang X, et al MRI-based radiomics signature for pretreatment prediction of pathological response to neoadjuvant chemotherapy in osteosarcoma: a multicenter study. Eur Radiol. 2021;31:7913-7924. 33825032 10.1007/s00330-021-07748-6

[37] Subramanian I , Verma S, Kumar S, Jere A, Anamika K. Multi-omics data integration, interpretation, and its application. Bioinform Biol Insights. 2020;14:1177932219899051. 32076369 10.1177/1177932219899051 PMC7003173

[38] Kim B-C , Kim J, Kim K, et al Preliminary radiogenomic evidence for the prediction of metastasis and chemotherapy response in pediatric patients with osteosarcoma using (18)F-FDF PET/CT, EZRIN and KI67. Cancers (Basel). 2021;13:2671. doi: 10.3390/cancers13112671. 34071614 PMC8198322

[39] Zöllner SK , Amatruda JF, Bauer S, et al Ewing sarcoma-diagnosis, treatment, clinical challenges and future perspectives. J Clin Med. 2021;10:1685. doi: 10.3390/jcm10081685. 33919988 PMC8071040

[40] Mankin HJ , Mankin CJ, Simon MA. The hazards of the biopsy, revisited. Members of the Musculoskeletal Tumor Society. J Bone Joint Surg Am. 1996;78:656-663. 8642021 10.2106/00004623-199605000-00004

[41] Chlorogiannis DD , Charalampopoulos G, Bale R, Odisio B, Wood BJ, Filippiadis DK. Innovations in image-guided procedures: unraveling robot-assisted non-hepatic percutaneous ablation. Semin Intervent Radiol. 2024;41:113-120. 38993597 10.1055/s-0044-1786724 PMC11236453

[42] Garnon J , Koch G, Tsoumakidou G, et al Ultrasound-guided biopsies of bone lesions without cortical disruption using fusion imaging and needle tracking: proof of concept. Cardiovasc Intervent Radiol. 2017;40:1267-1273. 28357575 10.1007/s00270-017-1638-9

[43] Groetz S , Wilhelm K, Willinek W, Pieper C, Schild H, Thomas D. A new robotic assistance system for percutaneous CT-guided punctures: initial experience. Minim Invasive Ther Allied Technol. 2016;25:79-85. 26902984 10.3109/13645706.2015.1110825

[44] Witkowska A , Petre EN, Moussa AM, et al Feasibility and safety of percutaneous CT-guided bone biopsies in patients with cancer using a patient-mounted robotic system: a retrospective analysis of 40 consecutive biopsies. J Vasc Interv Radiol. 2023;34:2174-2179. 37673400 10.1016/j.jvir.2023.08.040 PMC11260433

[45] Li H , Yan W, Zhao J, et al Navigate biopsy with ultrasound under augmented reality device: towards higher system performance. Comput Biol Med. 2024;174:108453. 38636327 10.1016/j.compbiomed.2024.108453

[46] Trojak M , Stanuch M, Kurzyna M, Darocha S, Skalski A. Mixed reality biopsy navigation system utilizing markerless needle tracking and imaging data superimposition. Cancers (Basel). 2024;16:1894. doi: 10.3390/cancers16101894. 38791972 PMC11119171

[47] Chacko R , Davis MJ, Levy J, LeBoeuf M. Integration of a deep learning basal cell carcinoma detection and tumor mapping algorithm into the Mohs micrographic surgery workflow and effects on clinical staffing: a simulated, retrospective study. JAAD Int. 2024;15:185-191. 38651039 10.1016/j.jdin.2024.02.014 PMC11033206

[48] Perivolaris A , Adams-McGavin C, Madan Y, et al Quality of interaction between clinicians and artificial intelligence systems. A systematic review. Future Healthc J. 2024;11:100172. 39281326 10.1016/j.fhj.2024.100172 PMC11399614

[49] Lennartz S , Dratsch T, Zopfs D, et al Use and control of artificial intelligence in patients across the medical workflow: single-center questionnaire study of patient perspectives. J Med Internet Res. 2021;23:e24221. 33595451 10.2196/24221 PMC7929746

[50] Labkoff S , Oladimeji B, Kannry J, et al Toward a responsible future: recommendations for AI-enabled clinical decision support. J Am Med Inform Assoc. 2024;31:2730-2739. 39325508 10.1093/jamia/ocae209 PMC11491642

[51] Jiang B , Mu Q, Qiu F, et al Machine learning of genomic features in organotropic metastases stratifies progression risk of primary tumors. Nat Commun. 2021;12:6692. 34795255 10.1038/s41467-021-27017-w PMC8602327

[52] He F , Xie L, Sun X, et al A scoring system for predicting neoadjuvant chemotherapy response in primary high-grade bone sarcomas: a multicenter study. Orthop Surg. 2022;14:2499-2509. 36017768 10.1111/os.13469 PMC9531107

[53] Hsieh TC , Liao CW, Lai YC, Law KM, Chan PK, Kao CH. Detection of bone metastases on bone scans through image classification with contrastive learning. J Pers Med. 2021;11:1248. doi: 10.3390/jpm11121248. 34945720 PMC8708961

[54] Tomaszewski MR , Gillies RJ. The biological meaning of radiomic features. Radiology. 2021;298:505-516. 33399513 10.1148/radiol.2021202553 PMC7924519

[55] Xu Z , Niu K, Tang S, et al Bone tumor necrosis rate detection in few-shot X-rays based on deep learning. Comput Med Imaging Graph. 2022;102:102141. 36446309 10.1016/j.compmedimag.2022.102141

[56] Ho DJ , Agaram NP, Jean M-H, et al Deep learning-based objective and reproducible osteosarcoma chemotherapy response assessment and outcome prediction. Am J Pathol. 2023;193:341-349. 36563747 10.1016/j.ajpath.2022.12.004 PMC10013034

[57] Lyu HG , Haider AH, Landman AB, Raut CP. The opportunities and shortcomings of using big data and national databases for sarcoma research. Cancer. 2019;125:2926-2934. 31090929 10.1002/cncr.32118 PMC6690764

[58] Tseng T-E , Lee C-C, Yen H-K, et al International validation of the SORG machine-learning algorithm for predicting the survival of patients with extremity metastases undergoing surgical treatment. Clin Orthop Relat Res. 2022;480:367-378. 34491920 10.1097/CORR.0000000000001969 PMC8747677

[59] van Leeuwen KG , Schalekamp S, Rutten M, van Ginneken B, de Rooij M. Artificial intelligence in radiology: 100 commercially available products and their scientific evidence. Eur Radiol. 2021;31:3797-3804. 33856519 10.1007/s00330-021-07892-z PMC8128724

[60] Regulation (EU) 2024/1689 of the European Parliament and of the Council of 13 June 2024 laying down harmonised rules on artificial intelligence and amending Regulations (EC) No 300/2008, (EU) No 167/2013, (EU) No 168/2013, (EU) 2018/858, (EU) 2018/1139 and (EU) 2019/2144 and Directives 2014/90/EU, (EU) 2016/797 and (EU) 2020/1828 (Artificial Intelligence Act) (Text with EEA relevance), (2024).

[61] von Schacky CE , Wilhelm NJ, Schäfer VS, et al Development and evaluation of machine learning models based on X-ray radiomics for the classification and differentiation of malignant and benign bone tumors. Eur Radiol. 2022;32:6247-6257. 35396665 10.1007/s00330-022-08764-w PMC9381439

